# Visual Feature Integration of Three Attributes in Stimulus-Response Mapping Is Distinct From That of Two

**DOI:** 10.3389/fnins.2019.00035

**Published:** 2019-02-13

**Authors:** Mizuki Furutate, Yumiko Fujii, Hiromi Morita, Masahiko Morita

**Affiliations:** ^1^Graduate School of Systems and Information Engineering, University of Tsukuba, Tsukuba, Japan; ^2^Graduate School of Library, Information and Media Studies, University of Tsukuba, Tsukuba, Japan; ^3^Faculty of Library, Information and Media Science, University of Tsukuba, Tsukuba, Japan; ^4^Faculty of Engineering, Information and Systems, University of Tsukuba, Tsukuba, Japan

**Keywords:** feature integration, binding problem, stimulus-response mapping, visual attention, object representation

## Abstract

In the human visual system, different attributes of an object are processed separately and are thought to be then temporarily bound by attention into an integrated representation to produce a specific response. However, if such representations existed in the brain for arbitrary multi-attribute objects, a combinatorial explosion problem would be unavoidable. Here, we show that attention may bind features of different attributes only in pairs and that bound feature pairs, rather than integrated object representations, are associated with responses for unfamiliar objects. We found that in a mapping task from three-attribute stimuli to responses, presenting three attributes in pairs (two attributes in each window) did not significantly complicate feature integration and response selection when the stimuli were not very familiar. We also found that repeated presentation of the same triple conjunctions significantly improved performance on the stimulus-response task when the correct responses were determined by the combination of three attributes, but this familiarity effect was not observed when the response could be determined by two attributes. These findings indicate that integration of three or more attributes is a distinct process from that of two, requiring long-term learning or some serial process. This suggests that integrated object representations are not formed or are formed only for a limited number of very familiar objects, which resolves the computational difficulty of the binding problem.

## Introduction

The human visual system is considered to process different visual attributes, such as shape, color, motion, and texture separately in different modules (Livingstone and Hubel, 1987). The integration of these distinct attributes to produce a unified percept and specific response is known as the binding problem (von der Malsburg, 1981; Treisman, 1996), one of the most important open problems in cognitive psychology and neuroscience. One main reason for the difficulty of this problem is the explosion of feature combinations, that is, the fact that the number of possible combinations of features of all attributes is extremely large. This problem is critical not only for the “cardinal cell” concept, which hypothesizes that all attributes are integrated via converging hard-wired connections into an integrated representation, but also for the concept of binding via synchronous firing of neurons (von der Malsburg, 1981; Singer and Gray, 1995), because this requires as many synchrony detectors as the number of feature combinations (Shadlen and Movshon, 1999).

Psychological studies (Luck and Vogel, 1997; Treisman, 1999; Wolfe and Cave, 1999) show that there exists a mechanism that integrates arbitrary combinations of features. According to the standard theory of feature integration (Treisman and Gelade, 1980), when attention is focused on an object, all attributes of the object are rapidly bound into a unified representation for higher cognitive processing (Treisman, 1988; Kahneman et al., 1992), which we refer to as the all-attribute model. However, no neural mechanisms have been found for such binding that are free from the combinatorial explosion problem. A clue to resolving this conflict may be that psychological evidence supporting the existence of feature binding does not require the existence of unified representations of all attributes. In fact, most studies of attentional binding have used two-attribute stimuli, and no studies have confirmed that three or more attributes are directly bound into unified representations. Furthermore, Hommel (1998) reported that only two-way interactions between feature-repetition effects were observed in a prime-probe stimulus-response (SR) task, suggesting that temporary binding may be binary, and that an object representation may comprise a loosely connected, distributed network of pairwise bindings rather than a unitary structure (Hommel and Colzato, 2004).

Accordingly, we hypothesized that attention can bind only pairs of attributes and that unified representations of three or more attributes are not formed (the “no-triplet hypothesis”), except perhaps in the case of a limited number of familiar objects. Based on this hypothesis, Morita et al. (Morita et al., 2010) developed a paired-attribute model, in which cognitive processes are based on multiple representations of paired attributes and their interactions, and discovered a new illusion arising from erroneous integration of attribute pairs, consistently with the model’s prediction. Moreover, Ishizaki et al. (2015) showed that learning and performance for SR tasks were more difficult when three attributes of the stimulus determined the correct response (Triple condition) than when two attributes did (Double condition), suggesting that bound feature pairs, rather than object representations, are associated with responses.

The results of the study by Ishizaki et al. support not only the paired-attribute model but also the no-triplet hypothesis, because the task was designed such that integration of multiple attributes was necessary. It seems unlikely that integrated representations of three attributes existed but were not used for such a task. To explain this in more detail, let us assign S_1_ and S_2_ as shape features, C_1_ and C_2_ as color features, and S*_i_*C*_j_* as the conjunction of S*_i_* and C*_j_*. If stimuli S_1_C_1_ and S_1_C_2_ are mapped to response R_1_, and stimuli S_2_C_1_ and S_2_C_2_ to response R_2_, SR mapping is easily achieved by associating S_1_ with R_1_ and S_2_ with R_2_. It is impossible, however, to associate stimuli S_1_C_1_ and S_2_C_2_ with response R_1_ and stimuli S_2_C_1_ and S_1_C_2_ with response R_2_, without integrating shape and color. Similarly, we can design a mapping between triple conjunctions and responses so that integration of three attributes is required.

In contrast, ordinary object recognition, visual search, or short-term memory tasks do not in principle require integration of attributes, because the tasks can be solved by comparing features for each attribute and integrating the comparison results; thus, experiments using such tasks cannot provide compelling evidence against the existence of integrated object representations. Accordingly, investigating the mapping process of multi-attribute stimuli to responses is critical to elucidate the representation underlying not only decision making, but also other various cognitive processes.

In the present study, we extended the previous study by Ishizaki et al. to obtain additional convincing evidence for the no-triplet hypothesis. Specifically, we performed the following two experiments using SR mapping tasks.

In Experiment 1, we tested a prediction derived from the paired-attribute model. In the previous study, spatially separated presentations of two or three attributes considerably complicated the SR task, although they did not markedly affect the target detection task, which does not require feature integration and response selection (Ishizaki et al., 2015). This indicates that feature integration and response selection became more difficult because separately presented features were not automatically bound by attention. The all-attribute model predicts that the same will occur if three attributes are presented separately in pairs (paired presentation), i.e., the SR task will be more complicated than the target detection task. However, according to the paired-attribute model, a three-attribute stimulus, say a red lattice-patterned circle, is represented by three attribute pairs—red circle, lattice-patterned circle, and red lattice pattern—which are separately associated with a response. This association process would be the same when three two-attribute stimuli are presented, and thus paired presentation will not affect feature integration and response selection. Accordingly, the paired-attribute model predicts that the paired presentation will not complicate the SR task more than the target detection task.

In Experiment 2, we examined the effect of stimulus familiarity on the SR mapping task. The no-triplet hypothesis does not exclude the integrated object representations for a limited number of familiar objects, implying that repeated presentation of the same feature combinations may promote their integration. The all-attribute model predicts that the familiarity effect will not appear or will appear independently of the number of attributes that need to be integrated if all attributes are presented as a single stimulus; the effect may more clearly appear when attributes are presented individually or in pairs so that the attributes cannot be bound by attention. In contrast, the paired-attribute model predicts that the familiarity effect will not appear strongly with the Double condition because even unfamiliar feature pairs can be quickly bound by attention but may appear more clearly with the Triple condition because integration of three attributes would require long-term learning. Thus, we compared familiar and unfamiliar stimuli with participants performing a familiarization task on the first day and a SR task on the following day.

## Materials and Methods

### Ethics Statement

This study was approved by the Ethical Committee of the Faculty of Library, Information and Media Science, University of Tsukuba, Japan, and was conducted in accordance with the Code of Ethics and Conduct of the Japanese Psychological Association. Written informed consent was obtained from all participants.

### Experiment 1

**FIGURE 1:**
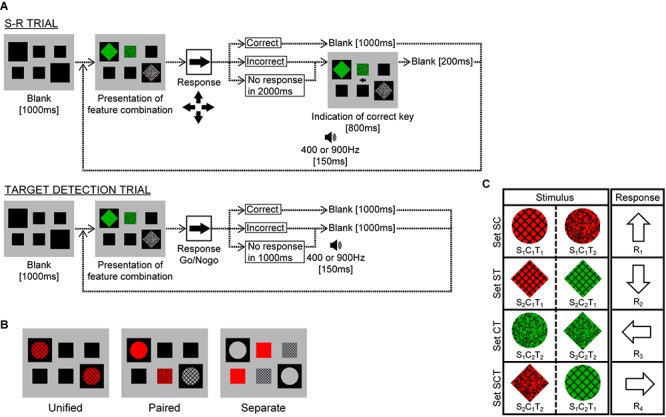
Experimental paradigm for Experiment 1. **(A)** Schematic procedure for stimulus-response (SR) and target detection trials. **(B)** Stimulus display. In the Unified, Paired, and Separate conditions, six features were presented in two, four, or six windows, respectively. Participants were instructed to respond as quickly and accurately as possible to the stimulus presented. **(C)** Correspondence between stimuli and responses in SR trials. The two stimuli comprising sets SC, ST, or CT differed only in texture, color, or shape, respectively, and corresponded to the same response key, whereas those in set SCT differed in all attributes.

The participants included 17 (7 male and 10 female) students with normal or corrected-to-normal vision. They were all paid volunteers who were uninformed of the experimental purpose. Participants viewed a CRT display from a distance of 114.5 cm in a dark room and responded by pressing a numerical keypad and performed SR trials and target detection trials (Figure 1A).

The display screen was gray (9.0 cd/m^2^), subtending 7.1 × 5.7° of visual angle, and had two large (1.9°) and four small (1.2°) square windows filled in black (Figure 1B). Stimuli were generated by combining two shapes (circle and diamond), two colors (red and green) with equal luminance (6.4 cd/m^2^), and two textures (lattice and random hashed lines) with equal average luminance (3.7 cd/m^2^) (Figure 1C). These features were common to all participants, but the mapping from feature combinations to response keys varied (counterbalanced across participants).

In each SR trial, after a blank screen showing only the presentation windows, one of the eight feature combinations was presented in the windows. Participants were instructed to select one of the four arrow keys and press it as quickly and accurately as possible. If the response was correct, the stimulus disappeared, and the next trial started with a 1000 ms blank screen; however, if the response was incorrect or no key was pressed within 2000 ms, a 400 Hz (incorrect) or 900 Hz (timeout) buzzer sounded for 150 ms and an arrow indicating the correct key was presented for 800 ms, after which the next trial started with a 200 ms blank screen.

In target detection trials, one of the eight feature combinations was designated as the target. Participants were requested to press a response key as quickly and accurately as possible when the target was presented in any presentation manner. If participants responded incorrectly to a non-target stimulus, a 400 Hz buzzer sounded, and if participants did not respond to the target within 1000 ms, a 900 Hz buzzer sounded. Simultaneously, with a correct response or a buzzer sound, the stimulus disappeared and the next trial started immediately.

There were three conditions: “Unified,” “Paired,” and “Separate.” In the Unified condition, two three-attribute stimuli were presented in two large windows (Figure 1B, left panel). These two stimuli were identical in most cases (10/11), and participants were requested to press one of the response keys as quickly and accurately as possible. Occasionally (1/11), however, the two objects were different in shape, in which case the participants were instructed not to press any key, indicating the need to attend to both windows. In the Paired condition, shape-color and shape-texture stimuli were presented inside the two large windows, and a color-texture stimulus was presented to fill the upper or lower (randomly selected) middle window (Figure 1B, middle panel). The participants were requested to press a key according to the combination of three attributes, except on occasional trials (1/11) when the shapes in the two large windows were different. In the Separate condition, the shape was presented inside the two large windows, the color was presented to fill the upper middle and the lower left small windows, and the texture was presented to fill the upper right and the lower middle small windows (Figure 1B, right panel). The participants were requested to press a key in the same way as in the Paired condition.

The mapping from the feature combinations to response keys is illustrated in Figure 1C, where the combination of shape S*_i_*, color C*_j_*, and texture T*_k_* is denoted as S*_i_*C*_j_*T*_k_* (*i*, *j*, *k* = 1 or 2). In set SC, for example, the combination presented was S_1_C_1_T_1_ or S_1_C_1_T_2_, and these were mapped to R_1._ Thus, the correct response was determined by shape and color but did not depend on texture. Similarly, the correct response did not depend on color and shape in sets ST and CT, respectively. In contrast, the three attributes were all critical in set SCT. One of the stimuli in sets SC, ST, and CT was presented as the “Double” condition, and either stimulus in set SCT was presented as the “Triple” condition. Combining these two conditions with three presentation conditions created six cases, which are denoted as Double-Unified, Triple-Paired, etc.

In each SR trial, one of the eight feature combinations and one of three presentation manners were pseudo-randomly selected, the stimulus display was presented, and the participant responded to it. The participants first performed 24 practice trials and 10 blocks of experimental trials for the SR task. Each block comprised 240 (8 × 3 × 10) SR trials, in which each feature combination appeared in each presentation manner 10 times, and 24 “catch” trials in which the shapes presented in the two large windows were different. The pseudo-random order of the stimuli was predetermined, which was constrained by two different stimuli in the same set (corresponding to the same response key) that were never presented in consecutive trials so that participants could not easily comprehend the mapping to a specific response.

Next, the participants performed 24 practice trials and one block of experimental trials for the target-detection task, in which one block comprised 240 (8 × 3 × 10) target-response trials and 24 catch trials, with the target appearing 30 times.

### Experiment 2

The participants were 18 students (5 male and 13 female) with normal or corrected-to-normal vision. They were all paid volunteers, who were uninformed of the experimental purpose and did not participate in Experiment 1. They performed a familiarization task on the first day and a SR task on the following day. The experimental environment was the same as that in Experiment 1.

**FIGURE 2:**
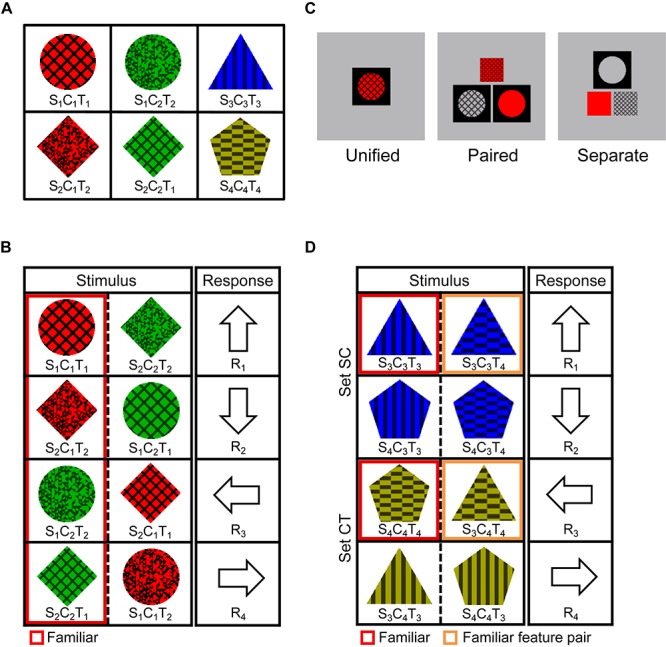
Experimental paradigm for Experiment 2. **(A)** Familiar stimuli. The six stimuli were each presented 700 times in the familiarization task, and then used as familiar stimuli or feature combinations in the SR task. **(B)** SR mapping for the Triple-conditions (Unified, Paired, and Separate). Stimuli surrounded by red lines were familiar, and the other stimuli were unfamiliar and never appeared in the familiarization task. **(C)** Stimulus displays for the Triple-conditions (Unified, Paired, and Separate). **(D)** SR mapping for the Double-Unified condition. Stimuli surrounded by orange lines were unfamiliar but contained a familiar feature pair by which the correct response could be determined.

In the familiarization task, the participants performed 42 blocks of target detection trials. For each block, one of the six stimuli shown in Figure 2A (fixed for all participants) was specified as the target, and the participants were instructed to press any key within 500 ms, only when the target was presented. Six three-attribute stimuli that differed from the target in only one attribute (shape, color, or texture) and would not be used in the SR task, were used as non-targets. If participants responded incorrectly to a non-target stimulus, a 400 Hz buzzer sounded, and if participants did not respond to the target within 500 ms, a 900 Hz buzzer sounded. The stimulus disappeared simultaneously with a correct response or a buzzer sound, and the next trial started immediately. Each block comprised 130 trials, in which the target appeared 100 times and non-targets appeared 30 (6 × 5) times in a random order.

After finishing one block, the participants proceeded to the next block, in which another stimulus was specified as the target. Six blocks, for six target stimuli, composed one cycle. Participants repeated seven cycles and viewed each of the six stimuli 700 times, which were used as the familiar stimuli in the SR task performed on the next day.

This task was similar to that in Experiment 1, except that the Triple and Double conditions and three presentation manners were not mixed in the same session. We also decreased the time limit when the average PCR in the previous block was over 90%, to create higher time pressure.

The experiment was performed under four conditions: Triple-Unified, Triple-Paired, Triple-Separate, and Double-Unified. The Triple-conditions (Unified, Paired, and Separate) were always performed in the order Separate–Paired–Unified to avoid the influence of viewing unfamiliar triple feature conjunctions on subsequent conditions. The Double-Unified condition was given first for half of the participants and last for the other half. Participants were requested to press the correct response key as quickly as possible within a time limit.

In the Triple-Unified condition, eight stimuli were mapped to four response keys, as shown in Figure 2B. The correct response was always determined by three attributes, and each response key corresponded to one familiar and one unfamiliar stimulus. Each trial started with a blank screen, which was gray (9.0 cd/m^2^), subtending 5.7 × 5.7° of visual angle, and had a single square window (1.9°) filled in black, after which one of the eight stimuli shown in Figure 2B was presented. If the response was correct, the stimulus disappeared, and the next trial started with a 1000 ms blank screen; however, if the response was incorrect or no key was pressed within the time limit, a 400 Hz (incorrect) or 900 Hz (timeout) buzzer sounded for 150 ms after the disappearance of the stimulus, and an arrow indicating the correct key was presented for 600 ms, after which the next trial started with a 400 ms blank screen.

The Triple-Paired and Triple-Separate conditions differed from the Triple-Unified condition only in that the blank screen had two (Paired) or one (Separate) large (1.9°) and one or two small (1.2°) square windows, and three attributes of the stimuli in Figure 2B were presented in pairs or separately in these windows (Figure 2C). The same combination was mapped to different keys among these three Triple conditions (e.g., S_1_C_1_R_1_ was mapped to R_1_, R_2_, and R_3_ in the Triple-Unified, -Paired, and -Separate conditions, respectively), and the participants performed the task in the order of separate, paired, and unified presentations, so that unfamiliar feature combinations would not become familiar.

The Double-Unified condition was the same as the Triple-Unified condition in the manner of stimulus presentation, but a different stimulus set (Figure 2D) was used. These eight stimuli were common to all participants, but three kinds of mapping were each applied to one third of the participants. That is, in addition to the mapping shown in Figure 2D, which consists of sets SC (the response is determined by shape and color) and CT (the response is determined by color and texture), mappings consisting of sets SC and ST (the response is determined by shape and texture) and consisting of sets CT and ST were used. In Figure 2D, the two stimuli surrounded by red lines were familiar triple conjunctions (Familiar case) and the others were unfamiliar triple conjunctions (Unfamiliar case), but each unfamiliar stimulus contained one familiar feature pair. We dealt with each case, in which the familiar feature pair was critical for determining the response (case Familiar feature pair, surrounded by orange lines), separately from the Unfamiliar case.

Participants first performed four blocks of practice trials in the Triple-Unified condition with a novel stimulus set, whose components were completely different from those for experimental trials, and performed 10 blocks of experimental trials in each condition. Each block comprised 80 (8 × 10) trials, in which each stimulus or feature combination appeared 10 times in a pseudo-random order, with the constraint that two different stimuli corresponding to the same response key were never presented in consecutive trials. The time limit was fixed to 2000 ms during the first five blocks, but it was thereafter controlled according to the average PCR in the previous block. Specifically, if the average PCR for all stimuli was over 90%, the time limit in the next block was shortened such that 90% of correct RTs were within it.

## Results

### Experiment 1

We analyzed data for 17 participants. For each participant and condition, the percentage of correct responses (PCR) of the SR trials for each block was calculated. Response times in “correct” trials were log-transformed and averaged within each block to calculate the mean response time (RT). In the same way, the mean target detection times (TDTs) were calculated from the response times in the target detection trials.

**FIGURE 3:**
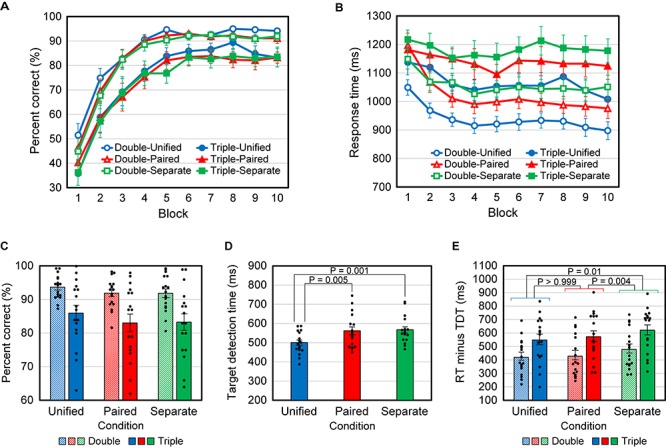
Results for Experiment 1. **(A)** Mean percent correct responses (PCR) versus block number. **(B)** Mean response time (RT) versus block number. **(C)** Mean PCR for blocks 6–10. All data points (*N* = 17) are plotted as dots. **(D)** Mean target detection time (TDT). **(E)** Mean response time minus target detection time (RT – TDT) for blocks 6–10. Error bars indicate SEM in all graphs.

Figure 3A,B show the time course, over the 10 blocks of PCR and RT, averaged over the 17 participants. We see that in any condition, the PCR increased and the RT decreased during the first five blocks, but were nearly constant thereafter. Therefore, to obtain stable responses, we analyzed only the data for the last half of the blocks (6 to 10).

The average PCR was analyzed using two-way repeated-measures ANOVA, with the number of critical attributes (conditions Double vs Triple) and the manner of presentation (conditions Unified vs Paired vs Separate) as factors. The main effect of attribute number was significant [*F*(1,16) = 31.0, *P* < 0.001], indicating that the mean PCR was significantly lower for the Triple conditions than for the Double conditions (Figure 3C). The main effect of the presentation manner was marginal [*F*(2,32) = 2.77, *P* = 0.078], likely because the correspondence between feature combinations and responses was common to all presentation manners. Also, the interaction [*F*(2,32) = 0.19, *P* = 0.83] was not found. Post-hoc multiple comparisons with Bonferroni correction were performed using two-tailed paired *t*-tests, and no significant differences were found between the Unified and Paired conditions (*P* = 0.20), between Unified and Separate (*P* = 0.24), and between Paired and Separate (*P* > 0.999).

The same analysis was applied to the average RT. The main effects of attribute number [*F*(1,16) = 32.1, *P* < 0.001] and presentation manner [*F*(2,32) = 54.7, *P* < 0.001] were significant, but their interaction was not [*F*(2,32) = 0.613, *P* = 0.55]. Post-hoc multiple comparisons with Bonferroni correction were performed using two-tailed paired *t*-tests, and significant differences were found between the Unified and Paired conditions (*P* < 0.001), between Unified and Separate (*P* < 0.001), and between Paired and Separate (*P* < 0.001).

TDTs were tested using repeated-measures ANOVA with three levels (Unified, Paired, and Separate), and a significant main effect was found [*F*(2,32) = 14.3, *P* < 0.001]. *Post hoc* multiple comparisons with Bonferroni correction were performed using two-tailed paired *t*-tests. Significant differences were found between the Unified and Paired conditions (*P* = 0.005) and between Unified and Separate (*P* = 0.001), but not between Paired and Separate (*P* > 0.999) (Figure 3D).

The differences in RT between presentation manners include the differences in the time required for perceiving features and the difference in TDT is considered to mainly reflect the difference in information acquisition time. Accordingly, we examined RT minus TDT (RT – TDT; Figure 3E). This value was calculated in each case (TDT is independent of the attribute number) for each participant and analyzed in the same way as PCR. We found that the main effects of attribute number [*F*(1,16) = 32.1, *P* < 0.001] and presentation manner [*F*(2,32) = 6.77, *P* = 0.004] were significant, but their interaction was not [*F*(2,32) = 0.613, *P* = 0.55]. Post-hoc multiple comparisons with Bonferroni correction (two-tailed paired *t*-test) were then performed, without distinction between the Double and Triple conditions because no significant interaction was found. The differences between the Unified and Separate conditions (*P* = 0.01) and between Paired and Separate (*P* = 0.004) were significant, but not between Unified and Paired (*P* > 0.999). Finally, we directly tested RT – TDT between the Triple-Unified and Triple-Paired cases and between the Double-Unified and Double-Paired cases, using two-tailed paired *t*-tests without Bonferroni correction, to confirm that no significant differences were found [*t*(16) = 0.908, *P* = 0.38 and *t*(16) = 0.431, *P* = 0.67, respectively].

The above results are summarized as follows: (1) The PCR was significantly smaller and the RT was significantly larger when triple conjunctions of attributes determined the response than when double conjunctions did. (2) The difference in RT between the Paired and Unified conditions was not significantly different from that in TDT, whereas the difference in RT between the Separate condition and the Unified or Paired condition was significantly larger than that in TDT.

### Experiment 2

We analyzed data from 14 participants whose PCR increased to more than 50% in all conditions. Data from four participants who failed to reach this criterion were excluded. For each participant and condition, PCRs for familiar and unfamiliar stimuli (also for familiar feature pairs in the Double-Unified condition) for each block were calculated. Similarly, RTs in correct trials were log-transformed and averaged to calculate RTs for familiar stimuli (and feature pairs) and unfamiliar stimuli.

**FIGURE 4:**
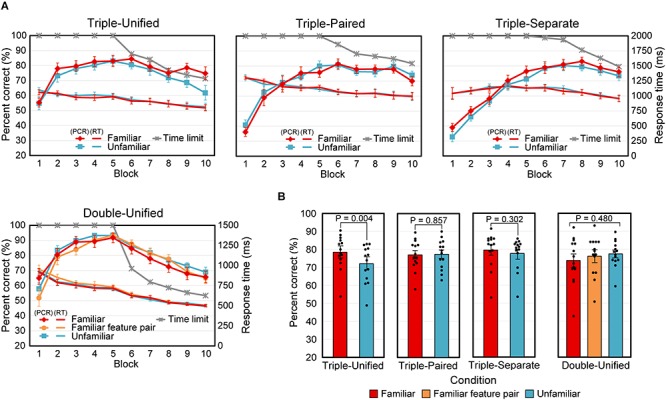
Results for Experiment 2. **(A)** Mean percent correct responses (PCR), response time (RT), and time limit versus block number. Error bars indicate SEM. **(B)** Mean PCR for the last two blocks. All data points (*N* = 14) are plotted as dots. The *P*-value for the Double-Unified condition indicates the significance level for the main effect in the ANOVA. Error bars indicate SEM.

Figure 4A shows the time courses of the mean PCR and mean RT, with the mean time limit, for the 14 analyzed participants. The curves for the familiar and unfamiliar cases almost overlapped, except the PCR curves in the last two blocks of the Triple-Unified condition.

The average PCR for the last half of the blocks (6 to 10) was tested (Figure 4B) using a two-tailed paired *t*-test for the Triple-conditions (Unified, Paired, and Separate). The differences between the Familiar and Unfamiliar cases were significant in the Triple-Unified condition [*t*(13) = 3.49, *P* = 0.004], but insignificant in the Triple-Paired [*t*(13) = −0.183, *P* = 0.86] and Triple-Separate [*t*(13) = 1.08, *P* = 0.30] conditions. A repeated-measures ANOVA with three levels (familiar stimuli, familiar feature pairs, and unfamiliar stimuli) was applied to the Double-Unified condition, and no significant main effect was found [*F*(2,26) = 0.755, *P* = 0.48]. We also analyzed RT in the same way, but did not find any significant differences (*P* > 0.46 for all comparisons).

In summary, the effect of stimulus familiarity was observed only when integration of three attributes was necessary and the stimuli were presented in a unified manner.

## Discussion

Conceivable models to explain how automatic binding by attention contributes to the SR mapping objects are as follows:

(A) Single-attribute model: Individual features are mapped to the response through learning, and attentional binding does not contribute to this process.
(B) All-attribute model: All (three) features of the object are automatically bound into a unitary representation, which is mapped to the response through learning.
(C) Paired-attribute model: Attention binds pairs of features, and multiple feature-pair representations are mapped to response through learning.

Our no triplet hypothesis, which states that attentional binding of arbitrary features occurs only between pairs of attributes and that triplets of attributes of an unfamiliar object are not directly integrated into a unified object representation, accords with the paired-attribute model for unfamiliar objects and conflicts with the all-attribute model for familiar and unfamiliar objects. Thus, let us examine these three models by comparing the experimental results.

First, the single-attribute model is not in accordance with the result of Experiment 1 in that RT – TDT was significantly longer for the separate presentation than for the paired or unified presentations, as the difference seems inexplicable without considering the contribution of attentional binding. For the same reason, the model appears inconsistent with the result of Experiment 2 in that the familiarity effect was observed only in the Triple-Unified condition.

Second, the all-attribute model is not in accordance with the results of Experiment 1 in that the PCR was lower and the RT was longer for the Triple condition than for the Double condition, because according to this model, any stimulus is mapped to the response via the object representation integrating three attributes in the Double and Triple conditions.

Additionally, the model appears inconsistent with the results in that separate presentation of stimuli increased RT – TDT compared to unified presentation but paired presentation did not. Although RT – TDT does not necessarily denote the time required for feature integration and response selection—as the response time is not a simple linear sum of time for detection, feature integration, and response selection—no significant difference in this value indicates that the difference in RT can be explained by the difference in information acquisition time. It may be natural that RT – TDT did not differ between unified and paired presentation for the Double condition, in which the correct response was determined by a feature pair; however, paired presentation did not increase it in the Triple condition either. This fact suggests that presenting two attributes at the same location contributes to feature integration and response selection, but presenting three attributes does not contribute more than that.

In addition, the all-attribute model is not in accordance with the result of Experiment 2 in that the effect of stimulus familiarity was observed in the Triple-Unified condition but not in the Double-Unified condition. Furthermore, the familiarity effect observed in the Triple-Unified condition disappeared in the Triple-Paired condition, implying that for familiar stimuli, paired presentation compared with unified presentation complicates feature integration and response selection in the Triple condition, whereas it does not for unfamiliar stimuli as indicated in Experiment 1. This is also difficult to explain with the all-attribute model.

In contrast, the above experimental results for unfamiliar stimuli are all as predicted or well explained by the paired-attribute model, which can also explain the result for the familiar stimuli. We therefore conclude that our results support the no-triplet hypothesis, indicating that bound feature pairs, rather than integrated object representations, are associated with responses for unfamiliar objects.

The no-triplet hypothesis allows that integrated representations of three or more attributes may exist for very familiar objects. However, this was not demonstrated by Experiment 2, because the familiarity effect was not observed during initial learning and because the task was obviously more difficult in the Triple condition than in the Double condition, even for familiar stimuli (although we cannot directly compare different conditions, the time limit for block 8 in the Triple-Unified condition and block 7 in the Double-Unified condition, for example, differed by more than 700 ms). If integrated representations of three attributes had been completely formed after the familiarization task, learning of the familiar stimuli would have been easier from the start, compared to learning of the unfamiliar stimuli, and performance would not have differed as much between the Triple and Double conditions.

The question, then, is how familiarity affected the feature integration process. According to the paired-attribute model, attributes at the same locations are integrated in pairs by attentional binding, and bound feature pairs are then associated with responses, with familiarity facilitating only the latter process. This model, however, is not in accordance with the result from Experiment 2 in that the familiarity effect was not observed in the paired presentation condition. Thus, a model with an additional path from individual features to responses, or a hybrid of the paired- and single-attribute models, would be more plausible. It should be noted that a distinct mechanism of feature integration using converging hardwired connections from lower-level modules for individual attributes is considered to exist independently of attentional binding (Hommel and Colzato, 2009; Vanrullen, 2009).

According to this two-path model, the results of our experiments can be explained as follows. If the stimulus is unfamiliar, only the first path—involving attentional binding—is available, and mapping to responses is easy in the Double condition. In the Triple condition, however, mapping from feature pairs to responses is complicated and not easily learned, so that “thinking,” or some serial process, would be involved in response selection. On the other hand, the second path is formed and available for very familiar stimuli, and is faster than the first path. This path does not necessarily make use of unified object representations, but may make use of types of integrated representations that do not completely correspond to individual objects. In the above experiment, complete object representations were not formed, presumably because the number of presentations was insufficient, or one day of familiarization was too short, or the familiarization task used did not require feature integration. In any case, if the component feature pairs are familiar but the stimulus is unfamiliar, or if the combination of three features is familiar but they are not presented in a unified manner, the second path would be available only partly, and the familiarity effect would disappear. However, this explanation is rather speculative, and further experiments (particularly with a longer period of familiarization) will be needed.

## Conclusion

In conclusion, the results of the present study indicate that in the mapping of multi-attribute visual stimuli to responses, feature integration of two attributes and of three attributes are distinct processes, in that the former is easy and automatic, and is not affected by the familiarity of feature conjunctions, whereas the latter is more difficult and is facilitated by repeated presentation of triple feature conjunctions. The results also provide additional evidence supporting the no-triplet hypothesis, which greatly facilitates solving the binding problem by avoiding the combinatory explosion problem, as previously discussed (Ishizaki et al., 2015). However, more evidence would be necessary to establish this hypothesis, because the possibility is not ruled out that attentional binding of three or more attributes may be used in some other cognitive process. It is also unclear how attention binds arbitrary features between pairs of attributes. Although answering this question requires further studies, we note that feature binding between pairs of attributes is computationally much easier than binding all attributes, and several biologically feasible mechanisms may be responsible, such as mutual modulation between neuronal populations encoding different attributes (Morita et al., 2010).

## Data Availability

The datasets generated for this study are available on request to the corresponding author.

## Author Contributions

MM and MF designed the experiments. MF performed the experiments with assistance from YF. YF and HM analyzed the data and prepared the figures. MM wrote the manuscript.

## Conflict of Interest Statement

The authors declare that the research was conducted in the absence of any commercial or financial relationships that could be construed as a potential conflict of interest. The handling Editor declared a shared affiliation, though no other collaboration, with several of the authors MM, MF, YF, and HM.
